# Local inhibition of nitrergic activity in tenotomized rats accelerates muscle regeneration by increasing fiber area and decreasing central core lesions

**DOI:** 10.1590/1414-431X20165556

**Published:** 2017-02-20

**Authors:** A.D. Seabra, S.A.S. Moraes, E.J.O. Batista, T.B. Garcia, M.C. Souza, K.R.M. Oliveira, A.M. Herculano

**Affiliations:** 1 Laboratório de Neurofarmacologia Experimental, Instituto de Ciências Biológicas, Universidade Federal do Pará, Belém, PA, Brasil; 2 Núcleo de Medicina Tropical, Universidade Federal do Pará, Belém, PA, Brasil

**Keywords:** Nitric oxide, Muscle regeneration, Atrophy, Tendon, Tenotomy

## Abstract

Muscular atrophy is a progressive degeneration characterized by muscular proteolysis, loss of mass and decrease in fiber area. Tendon rupture induces muscular atrophy due to an intrinsic functional connection. Local inhibition of nitric oxide synthase (NOS) by Nω-nitro-L-arginine methyl ester (L-NAME) accelerates tendon histological recovery and induces functional improvement. Here we evaluate the effects of such local nitrergic inhibition on the pattern of soleus muscle regeneration after tenotomy. Adult male Wistar rats (240 to 280 g) were divided into four experimental groups: control (n=4), tenotomized (n=6), vehicle (n=6), and L-NAME (n=6). Muscular atrophy was induced by calcaneal tendon rupture in rats. Changes in muscle wet weight and total protein levels were determined by the Bradford method, and muscle fiber area and central core lesion (CCL) occurrence were evaluated by histochemical assays. Compared to tenotomized (69.3±22%) and vehicle groups (68.1%±17%), L-NAME treatment induced an increase in total protein level (108.3±21%) after 21 days post-injury. A reduction in fiber areas was observed in tenotomized (56.3±1.3%) and vehicle groups (53.9±3.9%). However, L-NAME treatment caused an increase in this parameter (69.3±1.6%). Such events were preceded by a remarkable reduction in the number of fibers with CCL in L-NAME-treated animals (12±2%), but not in tenotomized (21±2.5%) and vehicle groups (19.6±2.8%). Altogether, our data reveal that inhibition of tendon NOS contributed to the attenuation of atrophy and acceleration of muscle regeneration.

## Introduction

Skeletal muscle atrophy is characterized by a loss of muscle mass associated with a diverse set of stressor events involving the musculoskeletal system, including tendon rupture. Indeed, disruption of tendon tissue induced by vocational or recreational activity can induce severe muscle atrophy, mainly during the process of tendon tissue recovery (1,2). Among experimental models, tenotomy has been used as a rapid inducer of muscle atrophy (3).

A recent work demonstrated that unloading following tendon rupture affects muscle-related gene expression (4). It is well documented that tenotomy has an immediate impact, with biochemical, morphological and functional muscle changes (5–8). Within a few days of Achilles tenotomy, areas of focal myofibrillar dissolution within soleus muscle fibers are observed, revealing the occurrence of central core lesions (CCLs) (6,9). CCLs are defined by the presence of a peripheral zone of normal appearance and a central zone characterized by myofibril misalignment and mitochondrial edema, as observed in cross-sections of muscle fibers (10).

A prolonged repair process follows tendon rupture, which is characterized by the production and release of several cytokines and growth factors, neuropeptides and other molecules, including nitric oxide (NO) (11,12). NO is an inorganic free radical with a diversity of physiological functions, synthesized from the amino acid L-arginine by three isoforms of the NO synthase enzyme (NOS; e.g., NOSI, NOSII, and NOS III) (13,14).

After tendon injury, all isoforms of NOS are found up-regulated in tendon tissue (15), and studies have implicated NO as an important molecule in tendon repair (12,16). However, side effects involving motor palsies, dyspnea and death may occur due to NO systemic modulation (16).

We have recently demonstrated that local NOS inhibition in a model of tendon rupture accelerates histological and functional recovery in murine Achilles tendon (17). Nevertheless, whether the effects of NOS inhibition on injured tendon might also be beneficial for muscle regeneration has not been evaluated. Considering that muscles and tendons are functionally integrated and that the nitrergic system plays a critical role in both tissues, we aimed to evaluate the biochemical and morphological parameters of muscle regeneration after tendon NOS inhibition in tenotomized rats (18,19).

## Material and Methods

### Animals

Adult male Wistar rats (240 to 280 g) were housed in polyacrylic cages with controlled temperature and lighting (21±2°C; 12/12 h light-dark cycle). Access to food and water was *ad libitum*. All experimental procedures were performed in accordance with the National Institutes of Health Guidelines for the Care and Use of Laboratory Animals and approved by the Animal Research Ethics Committee (#UFPA/BIO021-11). All efforts were made to minimize both the number of animals used and their suffering.

### Experimental groups

To evaluate whether tendon recovery induced by nitrergic blockage on tendon tissue is also able to induce soleus muscle recovery, we randomly divided the animals into four experimental groups: 1) control group (n=4), rats without injury or treatment; 2) tenotomized group (n=6), rats with injury but without treatment; 3) vehicle group (n=6), rats submitted to tenotomy that received 100 μL of 0.9% NaCl; 4) L-NAME group (n=6), injured rats that received 100 μL of 5 mM Nω-nitro-L-arginine methyl ester (L-NAME), a non-selective inhibitor of NOS (Sigma, USA). The treatments consisted of local injection of saline or L-NAME into the paratendinous region every 2 days after injury with a 26-gauge needle. The rats were supervised daily and weighed before tenotomy, as well as at 7, 14, and 21 days post-injury.

### Experimental tenotomy

Rats were intraperitoneally anesthetized with 10% ketamine hydrochloride (80 mg/kg) and 2% xylazine (12 mg/kg). Experimental tenotomy was performed in the right hind limb under aseptic conditions. The tendon was exposed through a midline skin incision posteriorly at the ankle. Before tendon rupture, a suture in accordance to the Kessler method with few modifications was made (20). Afterwards, the tendon was totally cut from 0.5 cm above the calcaneal insertion followed by tendon suture finalization and immediate skin sutures. No immobilization or movement restriction was utilized. After 14 or 21 days post-injury, all animals were sacrificed by decapitation and the right soleus muscles (about 2 cm length) were dissected, carefully removed and immediately weighed (wet weight).

### Total protein assay

Samples of soleus muscle were mechanically dissociated in phosphate buffered saline and an aliquot was used to determine the total protein content by the Bradford method, as described previously (21). Bovine serum albumin was used to obtain a protein standard curve. Total protein content was normalized per muscle wet weight and the values are reported as percent of the control.

### Histochemical evaluation

Soleus muscle samples were immersed in the optimal cutting temperature medium, frozen in liquid nitrogen, and cryosectioned at –24°C for histochemical analysis (22). Muscle serial cross-sections (20 µm) were collected on gelatin-coated glass slides. Sections were hydrated through graded alcohols and stained with hematoxylin-eosin (H&E). To determine fiber area and the percentage of fibers with CCL, cross-sections were photographed with a charge-coupled device camera (Moticam 2500, Switzerland) mounted on a light microscope (Nikon Eclipse 50i, Japan). Photomicrographs were employed for qualitative analysis and measurements of fiber diameter. A total of 200 fibers per animal were analyzed using the ImageJ¯ 1.47v software (National Institutes of Health, USA), as previously described (7).

### Statistical analysis

Data are reported as means±SD. Multiple comparisons were made by ANOVA followed by the Bonferroni test, and P<0.05 was considered to be statistically significant. All statistical analyses were performed using the software Prism 5.01v (GraphPad, USA).

## Results

### Body weight, muscle weight and protein levels

**Figure 1:**
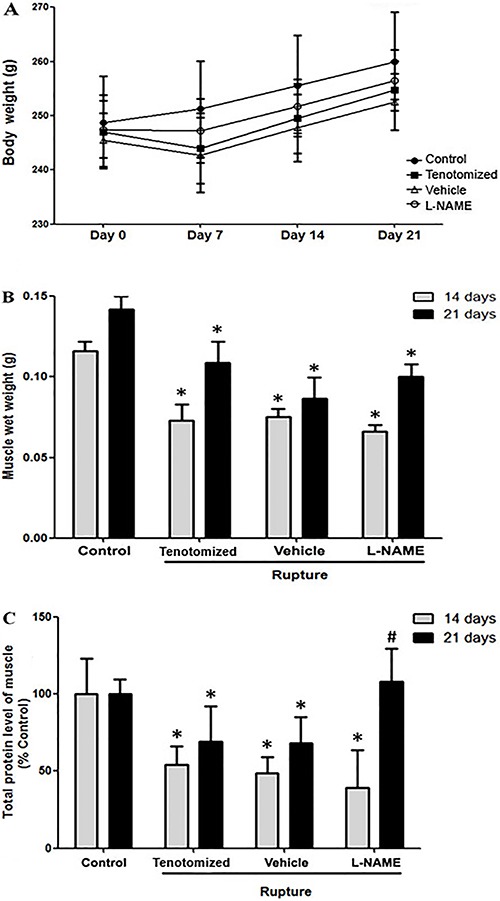
Effect of nitrergic inhibition on body weight, muscle wet weight and total protein levels of rats measured on days 14 and 21 after tenotomy. *A*, Body weight; no difference between groups was observed. *B*, Effect of nitrergic inhibition on soleus wet weight. *C*, Total protein levels of muscle normalized per muscle wet weight and the values are reported as percent of control. Data are reported as means±SD for n=4 (control) or n=6 rats (all other groups). *P<0.05 *vs* respective controls. ^#^P<0.05 *vs* tenotomized and vehicle on day 21 after tenotomy (ANOVA-Bonferroni).

Body weight was not different between control and injured rats (Figure 1A). Soleus muscle wet weight, however, was significantly reduced at both 14 (approximately 62% of control) and 21 (approximately 70% of control) days after tenotomy in all groups (Figure 1B).

Total protein levels from soleus muscle of tenotomized group were significantly reduced to 54.1±11.8% of control on day 14 and 69.3±22% of control on day 21 following tenotomy and repair (Figure 1C). Similar effects were observed in the vehicle group, with reduction to 48.6%±10.7% of control on day 14 and 68.1%±17% of control on day 21 after injury. No effect after treatment with L-NAME was observed on day 14 after tenotomy. Whereas, on day 21 after tenotomy, the total protein levels of the soleus muscle increased significantly to 108.3±21% of control in the L-NAME-treated group compared to tenotomized and vehicle groups, displaying similar levels to the control group (Figure 1C).

### Morphological and muscle fiber area analysis

**Figure 2:**
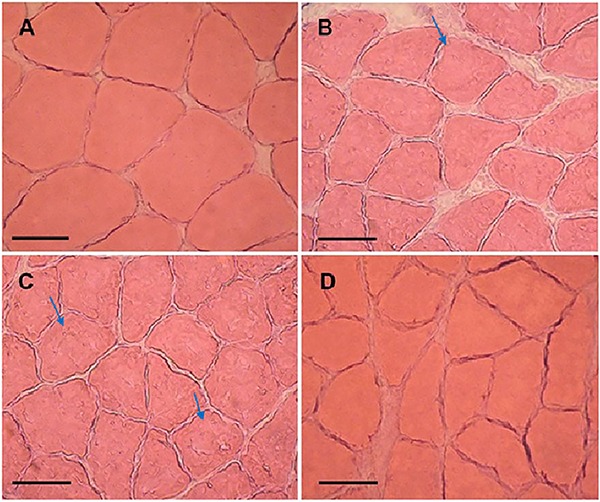
Morphological analysis of muscle fibers stained with H&E on day 21 post-injury. *A*, Control; *B*, tenotomized; *C*, vehicle, and *D*, L-NAME. Groups that underwent tenotomy, but no treatment with L-NAME (*B*, *C*) displayed slight morphological alterations in muscle fibers. Arrows show probable CCLs (non-classical morphology). Treatment with L-NAME induced morphological improvement of fibers (*D*). Scale bar: 200 μm; n≥4 rats/group.

**Figure 3:**
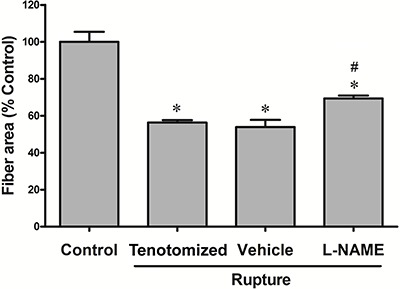
Quantification of muscle fiber areas. Muscle samples were obtained on day 21 after tenotomy from all experimental groups. About 200 fibers per animal were evaluated. Fiber area is reported as means±SD in percent of control for n≥4 rats/group. *P<0.05 *vs* control. ^#^P<0.05 *vs* tenotomized and vehicle (ANOVA-Bonferroni).

Microscopic evaluation of the soleus muscles suggested that the reduction of protein content was associated with morphological changes to the internal muscle fiber structures on day 21 after tenotomy (Figure 2). Muscle fibers from both tenotomized and vehicle groups displayed a pale staining and a halo of myofibril degeneration (Figure 2B and C, indicated by arrow). Such alterations resembled CCLs, but with a non-classical morphology. On the other hand, treatment with L-NAME (Figure 2D) induced a histological improvement, showing more intact fibers, similar to the control group (Figure 2A). We measured fiber area on day 21 post-injury as an index of atrophy and found that tendon rupture led to a significant reduction in myofibril area in tenotomized (56.3±1.3% of control) and vehicle groups (53.9±3.9% of control; Figure 3). Local treatment with L-NAME increased the fiber area to 69.3±1.6% of control compared to tenotomized and vehicle groups (P<0.05), even though this value still remained below control levels.

### Central core lesions

**Figure 4:**
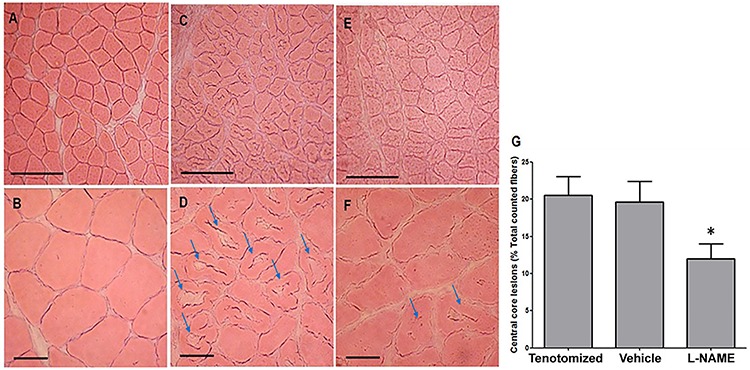
Analysis of central core lesion (CCL) occurrence on day 14 after injury. Control group (*A*, *B*), tenotomized group (*C*, *D*) and L-NAME group (*E, F*). Muscle fibers from the L-NAME group displayed remarkable histological alterations characteristic of CCL. Although CCLs were present in the L-NAME group, the occurrence of lesions was smaller than in the control group. Arrows: CCL in muscle fiber. Scale bar: 200 μm (*A*, *C*, *E*). Scale bar: 50 μm (*B*, *D*, *F*). n≥4 rats/group. *G*, Quantification of muscle fibers with CCL. A total of 180 fibers were evaluated from rats that underwent tenotomy (tenotomized, vehicle and L-NAME groups). Tenotomized and vehicle groups showed about 20% of muscle fibers with CCL. Treatment with L-NAME displayed a significant reduction of fibers with CCL (about 12%). Data are reported as means±SD. *P<0.05 *vs* tenotomized and vehicle (ANOVA-Bonferroni).

As CCLs were absent from muscle fibers on day 21 after tenotomy, we examined the effect of treatment with L-NAME on day 14 after tenotomy by staining cross-sections of muscle fibers with H&E and counting the number of CCLs. Injured groups showed a prominent morphological alteration of fiber structure with a high occurrence of CCLs compared to the control group (Figure 4A–D). Several fibers displayed a pale-stained central area of various shapes (suggesting a continuous degeneration of myofibrils) and an unremarkable peripheral zone. Nevertheless, treatment with L-NAME induced a reduction in the number of fibers with CCL (Figure 4E and F). A semi-quantitative analysis (Figure 4G) showed that the mean percentage of fibers with CCL in the L-NAME group was significantly lower than in tenotomized or vehicle groups (12±2% L-NAME *vs* 21±2.5% tenotomized or 19.6±2.8% vehicle group, P<0.05).

## Discussion

Here we demonstrated that local NOS inhibition in tenotomized rats induced biochemical and morphological recovery of soleus muscle. Treatment with L-NAME induced a significant increase of total protein level on day 21 after tenotomy preceded by a remarkable reduction in the number of fibers with CCL on day 14 after tenotomy.

It is well-known that NO displays a dual action on biological systems, leading to cytotoxic effects when in high concentrations and playing important physiological roles when in low concentrations (13,14). Therefore, the route for NOS inhibitor delivery must be carefully considered. Consistent with previous studies, we found that L-NAME has beneficial effects without influencing body weight gain, suggesting that such effects are due to local and not systemic outcomes of the NOS inhibitor (17,23).

Muscle mass change is one of the many metabolic alterations that may occur during muscle atrophy (24–26). Muscle mass and the maintenance of its functional capacity are controlled by the balance between protein synthesis and degradation. In accordance with previous published studies, we observed that muscle wet weight decreased significantly in injured animals on both days 14 and 21 after tenotomy (27,28). In addition, total protein levels from soleus muscle decreased by about 55 and 30% on days 14 and 21 after tenotomy, respectively. Treatment with L-NAME showed no effect on decrease of the muscle wet weight on days 14 and 21 after tenotomy, whereas local NOS inhibition on day 21 induced an increase in total protein level. It is likely that the high water content of muscle may limit a detectable change in the muscle wet weight between the groups (29).

It has been suggested that NO leads to the inhibition of type I collagen synthesis in fibroblasts (30). However, some studies differ as to the effects of NO on type I collagen synthesis by tendon cells, as well as its influence on cell adhesion (31,32). The use of NOS inhibitors appears to hinder the molecular pathways involved in atrophy that is triggered by NO release (33). Together, these findings suggest that inhibition of NO synthesis during tendon injury and consequent muscle atrophy are important for the repair and attenuation of atrophic process by preventing protein level decrease.

Protein degradation and loss of muscle mass are associated with the reduction of muscle fiber area, a hallmark of atrophy (10,24,34). We have observed morphological alterations and a significant decrease in fiber area on day 21 after tenotomy. The effect of L-NAME, although relatively modest on the fiber area, showed a remarkable histological recovery.

Moreover, a spontaneous reduction of CCLs also seems to occur; no area showing the classical morphology of CCL in muscle fibers was observed on day 21 after tenotomy, whereas CCLs were observed on day 14 after tenotomy, similar to those previously described (7,10,35). Interestingly, treatment with L-NAME induced a significant reduction in the number of fibers with CCL, suggesting that NOS inhibition can accelerate muscle recovery. Following tenotomy, the number of sarcomeres in series decreases due to fiber shortening, and this seems to be related to CCL formation (5,7,36). Such morphological alterations are closely associated with muscular functional deficit. For instance, NO production is increased during experimental Duchenne muscular dystrophy, leading to muscle force reduction (37). Our data suggest that L-NAME acted via two distinct mechanisms: 1) decreasing the number of CCLs and 2) increasing the total protein levels, resulting in morphological improvement, including in the fiber area.

Thus, local injection of L-NAME can help to prevent the development of muscle atrophy by hindering the biochemical and morphological changes that are typically observed in various models of tendon rupture, including decrease of muscle mass and protein levels, as well as the occurrence of CCL and reduction of muscle fiber area (10,25,27,34,36,38–40). However, further investigations are still required to reveal the mechanisms underlying the effects of NOS inhibition in tendon and how such effects reach the muscle area. In conclusion, our data suggest that morphological and biochemical improvements in tendon after local NOS inhibition are extended to muscle structure.

## References

[1] Padanilam TG (2009). Chronic Achilles tendon ruptures. Foot Ankle Clin.

[2] Schepsis AA, Jones H, Haas AL (2002). Achilles tendon disorders in athletes. Am J Sports Med.

[3] Bialek P, Morris C, Parkington J, St Andre M, Owens J, Yaworsky P (2011). Distinct protein degradation profiles are induced by different disuse
models of skeletal muscle atrophy. Physiol Genomics.

[4] Killian ML, Lim CT, Thomopoulos S, Charlton N, Kim HM, Galatz LM (2013). The effect of unloading on gene expression of healthy and injured
rotator cuffs. J Orthop Res.

[5] Jamali AA, Afshar P, Abrams RA, Lieber RL (2000). Skeletal muscle response to tenotomy. Muscle Nerve.

[6] Baewer DV, van Dyke JM, Bain JL, Riley DA (2008). Stretch reduces central core lesions and calcium build-up in
tenotomized soleus. Muscle Nerve.

[7] van Dyke JM, Bain JL, Riley DA (2012). Preserving sarcomere number after tenotomy requires stretch and
contraction. Muscle Nerve.

[8] Martin TD, Dennis MD, Gordon BS, Kimball SR, Jefferson LS (2014). mTORC1 and JNK coordinate phosphorylation of the p70S6K1
autoinhibitory domain in skeletal muscle following functional
overloading. Am J Physiol Endocrinol Metab.

[9] Baewer DV, Hoffman M, Romatowski JG, Bain JL, Fitts RH, Riley DA (2004). Passive stretch inhibits central corelike lesion formation in the
soleus muscles of hindlimb-suspended unloaded rats. J Appl Physiol.

[10] Abou Salem EA, Fujimaki N, Ishikawa H, Tashiro T, Komiya Y (2001). Morphological changes and recovery process in the tenotomized soleus
muscles of the rat. Arch Histol Cytol.

[11] Sharma P, Maffulli N (2006). Biology of tendon injury: healing, modeling and
remodeling. J Musculoskelet Neuronal Interact.

[12] Tomiosso TC, Nakagaki WR, Gomes L, Hyslop S, Pimentel ER (2009). Organization of collagen bundles during tendon healing in rats treated
with L-NAME. Cell Tissue Res.

[13] Moncada S, Higgs EA (1995). Molecular mechanisms and therapeutic strategies related to nitric
oxide. FASEB J.

[14] Moncada S, Palmer RM, Higgs EA (1991). Nitric oxide: physiology, pathophysiology, and
pharmacology. Pharmacol Rev.

[15] Lin J, Wang MX, Wei A, Zhu W, Murrell GA (2001). The cell specific temporal expression of nitric oxide synthase
isoforms during achilles tendon healing. Inflamm Res.

[16] Murrell GA, Szabo C, Hannafin JA, Jang D, Dolan MM, Deng XH (1997). Modulation of tendon healing by nitric oxide. Inflamm Res.

[17] Moraes SA, Oliveira KR, Crespo-Lopez ME, Picanco-Diniz DL, Herculano AM (2013). Local NO synthase inhibition produces histological and functional
recovery in Achilles tendon of rats after tenotomy: tendon repair and local NOS
inhibition. Cell Tissue Res.

[18] Roberts TJ (2002). The integrated function of muscles and tendons during
locomotion. Comp Biochem Physiol A Mol Integr Physiol.

[19] Kjaer M (2004). Role of extracellular matrix in adaptation of tendon and skeletal
muscle to mechanical loading. Physiol Rev.

[20] Barmakian JT, Lin H, Green SM, Posner MA, Casar RS (1994). Comparison of a suture technique with the modified Kessler method:
resistance to gap formation. J Hand Surg Am.

[21] Bradford MM (1976). A rapid and sensitive method for the quantitation of microgram
quantities of protein utilizing the principle of protein-dye
binding. Anal Biochem.

[22] Jozsa L, Balint BJ, Vandor E, Reffy A, Demel Z (1985). Recapillarization of tenotomized skeletal muscles after delayed tendon
suture. I. Experimental study. Res Exp Med.

[23] Ross RM, Wadley GD, Clark MG, Rattigan S, McConell GK (2007). Local nitric oxide synthase inhibition reduces skeletal muscle glucose
uptake but not capillary blood flow during in situ muscle contraction in
rats. Diabetes.

[24] Maxwell LC, Enwemeka CS (1992). Immobilization-induced muscle atrophy is not reversed by lengthening
the muscle. Anat Rec.

[25] Jackman RW, Kandarian SC (2004). The molecular basis of skeletal muscle atrophy. Am J Physiol Cell Physiol.

[26] Powers SK, Smuder AJ, Criswell DS (2011). Mechanistic links between oxidative stress and disuse muscle
atrophy. Antioxid Redox Signal.

[27] Jakubiec-Puka A, Catani C, Carraro U (1992). Myosin heavy-chain composition in striated muscle after
tenotomy. Biochem J.

[28] Giger JM, Bodell PW, Zeng M, Baldwin KM, Haddad F (2009). Rapid muscle atrophy response to unloading: pretranslational processes
involving MHC and actin. J Appl Physiol.

[29] Mantle BL, Hudson NJ, Harper GS, Cramp RL, Franklin CE (2009). Skeletal muscle atrophy occurs slowly and selectively during prolonged
aestivation in *Cyclorana alboguttata* (Gunther
1867). J Exp Biol.

[30] Dooley A, Gao B, Shi-Wen X, Abraham DJ, Black CM, Jacobs M (2007). Effect of nitric oxide and peroxynitrite on type I collagen synthesis
in normal and scleroderma dermal fibroblasts. Free Radic Biol Med.

[31] Xia W, Wang Y, Appleyard RC, Smythe GA, Murrell GA (2006). Spontaneous recovery of injured Achilles tendon in inducible nitric
oxide synthase gene knockout mice. Inflamm Res.

[32] Molloy TJ, Wang Y, Horner A, Skerry TM, Murrell GA (2006). Microarray analysis of healing rat Achilles tendon: evidence for
glutamate signaling mechanisms and embryonic gene expression in healing tendon
tissue. J Orthop Res.

[33] Suzuki N, Motohashi N, Uezumi A, Fukada S, Yoshimura T, Itoyama Y (2007). NO production results in suspension-induced muscle atrophy through
dislocation of neuronal NOS. J Clin Invest.

[34] Maxwell LC, Moody MR, Enwemeka CS (1992). Muscle atrophy continues after early cast removal following tendon
repair. Anat Rec.

[35] Baker JH (1985). The development of central cores in both fiber types in tenotomized
muscle. Muscle Nerve.

[36] Baker JH, Hall-Craggs EC (1980). Changes in sarcomere length following tenotomy in the
rat. Muscle Nerve.

[37] Li D, Yue Y, Lai Y, Hakim CH, Duan D (2011). Nitrosative stress elicited by nNOSmicro delocalization inhibits
muscle force in dystrophin-null mice. J Pathol.

[38] Tomanek RJ, Cooper RR (1972). Ultrastructural changes in tenotomized fast- and slow-twitch muscle
fibres. J Anat.

[B39] Wahlby L, Dahlback LO, Sjostrom M (1978). Achilles tendon injury. II. Structure of tenotomized rabbit crural
muscles after primary and delayed tendon suture. Acta Chir Scand.

[40] Barry JA, Cotter MA, Cameron NE, Pattullo MC (1994). The effect of immobilization on the recovery of rabbit soleus muscle
from tenotomy: modulation by chronic electrical stimulation. Exp Physiol.

